# How Does Emotional Intelligence Make One Feel Better at Work? The Mediational Role of Work Engagement

**DOI:** 10.3390/ijerph15091909

**Published:** 2018-09-02

**Authors:** Natalio Extremera, Sergio Mérida-López, Nicolás Sánchez-Álvarez, Cirenia Quintana-Orts

**Affiliations:** 1 Faculty of Psychology, University of Málaga, 29071 Málaga, Spain; sergioml@uma.es (S.M.-L.); cquintana@uma.es (C.Q.-O.); 2 Deusto Stress Research, University of Deusto, 48007 Bilbao, Spain; nsa@deusto.es

**Keywords:** emotional intelligence, engagement, job satisfaction, multi-occupational sample

## Abstract

Although previous research has highlighted the association between emotional intelligence (EI) and job satisfaction, the underlying mechanisms remain relatively unexplored. To address this gap, this study examined employee engagement as a potential mediator of the association. A multi-occupational sample of 405 Spanish professionals completed the Wong Law Emotional Intelligence Scale, the Utrecht Work Engagement Scale and an Overall Job Satisfaction Scale as well as providing socio-demographic data. As expected, employees’ EI was positively related to engagement dimensions (vigour, dedication and absorption) as well as overall job satisfaction. Bootstrap estimates from multiple mediation analysis confirmed that employees’ perceived EI was indirectly associated with job satisfaction via vigour and dedication scores, even when controlling for the effects of socio-demographic variables. Similarly, the same pattern was found when multiple mediation was conducted for each EI dimension. Our study contributes to understanding of the processes involved in maintaining and enhancing positive attitudes at work, providing the first, encouraging evidence that work engagement play a role in the EI-job satisfaction link. Our results extend the EI literature by elucidating the pathways through which EI is linked to positive employee attitudes and suggests that intervention programs designed to bolster EI might prove effective at increasing job satisfaction.

## 1. Introduction

Employees’ feelings in the workplace and attitudes to their job vary. One of most well-known job attitude variables in the field of organisational behaviour is job satisfaction, because job satisfaction has the potential to influence how we behave at work [1]. Many studies have found a strong relationship between employee attitudes and workplace variables such as job performance, organizational citizenship behaviour and work withdrawal, amongst others [2]. Although there are multiple definitions of job satisfaction one of the most widely used is that proposed by Locke [3], who defined job satisfaction as a pleasurable or positive emotional state resulting from the appraisal of one’s job or job experiences. More recently, Judge et al. [4] defined job satisfaction as an evaluative state that expresses contentment with and positive feelings to the one’s job, including both cognitive (contentment) and affective components (positive feeling).

In the last two decades, there has been considerable attention paid to individual differences in job satisfaction in the organisational literature [2,4]. It has been established that individual differences in employee variables have impact on workplace satisfaction over and above job factors or organisational factors [5]. Consistent with this there is a growing body of evidence that an individual’s disposition influences his or her affective experiences at work [6]. Interest in the dispositional aspect of job satisfaction has focused on three specific theoretical constructs: positive and negative affectivity [7], the five-factor model of personality [8] and core self-evaluations [9]. A meta-analysis concluded that both positive and negative moods have similarly strong associations with job satisfaction (0.32) [10]. Another meta-analytic study concluded that four of the Big Five personality traits, namely neuroticism, conscientiousness, extraversion and agreeableness, are related to job satisfaction; all together, the Big Five traits show a moderate association with job satisfaction (0.41) [11]. Finally, a meta-analysis by Judge and Bono [12] concluded that core self-evaluation—i.e., people’s fundamental evaluations of their worth, competence, capabilities and functioning in their environment—also has a moderate association with job satisfaction (0.32). In general, these dispositional approaches of job satisfaction argue that employees’ affective disposition may influence their perception of emotionally significant events at work, which in turn may influence their job satisfaction [13]. Each dispositional approach has received accumulated empirical evidence, showing the incremental validity of affective dispositions over organisational characteristics in predicting job satisfaction. As Judge and Larsen [5] acknowledged, however, it is necessary to expand this new field of research including other personal resources that influence one’s perceptions of one’s workplace and hence one’s job satisfaction.

### 1.1. Emotional Intelligence and Job Satisfaction

The construct of emotional intelligence (EI) emerged as part of the “affective revolution” in organisational and work psychology [14]. EI is defined as a psychological resource composed of a set of abilities concerned with the processing of emotion-relevant information and it is one possible contributor to positive job attitudes and behaviours and, specifically, job satisfaction [15,16,17].

A substantial body of research has focused on emotional abilities as predictors of job performance and job satisfaction over and above classic well-known constructs such as personality traits and cognitive intelligence [15,18]. Mayer and Salovey [19] defined EI as a set of skills for perceiving, accessing and generating emotions in order to assist thought, understand emotions and emotional knowledge and regulate emotions in a considered way that will promote emotional and intellectual growth. Employees vary in their ability to process, understand and use emotional information in the workplace [20]. Kafetsios and Zampatekis [21] noted that there are a number of reasons why workers with high EI might experience higher job satisfaction. At the intrapersonal level, one would expect that individuals who understand their own moods and can use them effectively would have the skills and resources required to repair negative moods, regulate emotions, withstand workplace stress and increase job satisfaction. At the interpersonal level, one would expect individuals who are good at understanding and regulating the emotions of others to benefit from better interpersonal relationships and social networks and to increase the prevalence of positive mood in the workplace. Consistent with this idea, employees with high EI report more positive attitudes and behaviour in the workplace than their peers with lower EI, even when the influence of cognitive intelligence and personality traits is controlled [15,17,22].

In recent years, most research on EI and job satisfaction has focused on the direct associations between measures of EI and various job satisfaction indicators [23,24,25]. There is a new line of research examining potential affective workplace-related mediators of the relationship between EI and job satisfaction [21]. A recent meta-analysis concluded that positive and negative affect in the workplace mediate the link between EI and job satisfaction, with positive affect being the stronger mediator [16]. Affective event theory posits that emotions and affective disposition influence the relationships between dispositional factors and job satisfaction and workplace performance [6,26]. Objective working conditions influence job attitudes but employees’ affective state may also influence their perceptions and reactions to events at work. In the same way, researchers have argued that one affective-motivational state called work engagement might be an important mediator between the influence of personal resources and organisational outcomes [27,28,29,30].

### 1.2. Work Engagement as Potential Mediator of the EI-Job Satisfaction Link

Work engagement is a theoretical construct proposed by Maslach and Leiter [31] and later developed by Schaufeli et al. [32]; it is consistent with the ideas of the positive psychology movement. Work engagement is generally defined as a positive, fulfilling, work-related state of mind characterised by vigour, dedication and absorption [32]. In this context, vigour is defined as energy and resilience, that is, a willingness to invest effort in one’s job and to persist at work-related tasks. Dedication is characterised by strong involvement in one’s job and job-related enthusiasm, pride and inspiration. Finally, absorption involves being happily engrossed in one’s work, to the extent that time passes quickly and one has difficulty detaching oneself from one’s work. Engagement is the opposite of burnout, it is a persistent and pervasive affective–motivational state of work-related well-being that is not focused on any particular object, event, individual or behaviour [32]. Several researchers have reported negative relationships between engagement and burnout variables [33], as well as differences in the work-related antecedents and outcomes, including job satisfaction, of both theoretical constructs [34,35].

In summary, there is a robust association between EI and job satisfaction but so far, the potential role of engagement in this association has not been explored. We argue that EI may contribute to the affective component of job satisfaction by increasing engagement. Miao, Humphrey and Qian [16] proposed that EI may modify perceptions of and reactions to a wide variety of organisational events (e.g., high EI might result in more use of active strategies for coping with antagonistic work colleagues or positive reinterpretations of organisational stressors). As employees know how to regulate their own affective reactions in organisations and to act in ways that foster better interpersonal relationships with colleagues and supervisor, it might motivate them to exert more effort and feel more energy and pride at work. At the same time, as employees feel more positive states at work they might appraise one’s job experiences as more pleasurable, indicating higher job satisfaction [3,4]. This potential mechanism has been suggested but not tested until now.

### 1.3. Rationale for This Study

Our main objective was to examine the potential role of engagement in the EI-job satisfaction link. There are a number of reasons why work engagement might mediate this relationship. First, there is increasing empirical evidence of a positive association between EI and job satisfaction [16,18,21]. Second, there is mounting evidence that individuals with high EI report higher vigour, dedication and absorption [36,37,38]. Third, past studies have shown that work engagement is an important predictor of positive job attitudes, including job satisfaction [35,39,40]. Finally, because several studies have demonstrated that socio-demographic variables, such as age and gender, are associated with both EI [41,42] and job satisfaction [43,44], we controlled for age and gender effects in this study. Similarly, years of experience has been found to be an important predictor of job satisfaction [45,46], so we also controlled for years of experience in the mediation analyses.

**Figure 1:**
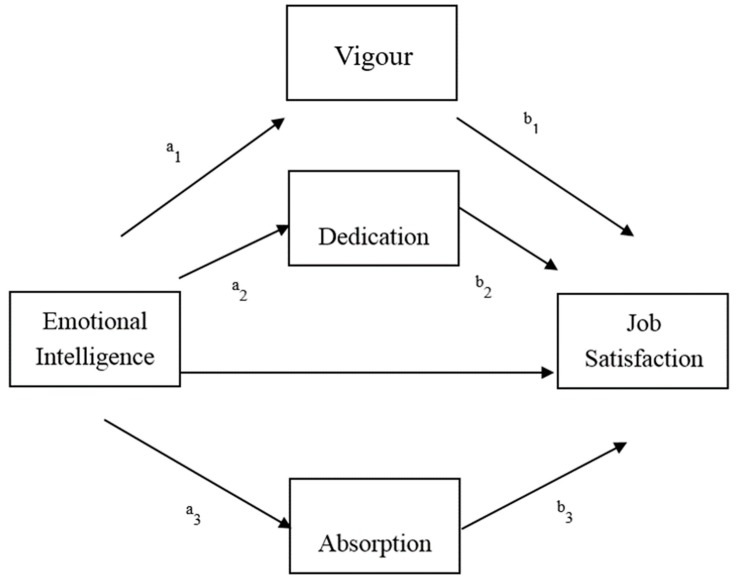
Proposed model of the role of work engagement dimensions in explaining job satisfaction. ** *p* < 0.01.

This study had two main objectives. First, we examined the relationships among EI, engagement levels and job satisfaction in a multi-occupational sample. Second, consistent with our proposed mediational model, we analysed if engagement dimensions (vigour, dedication and absorption) would mediate the relationship between EI and job satisfaction. On the basis of prior research, we hypothesised that EI would be positively associated with work engagement variables [36] and job satisfaction [24]. Prior research also suggested that we would find a positive association between engagement levels and job satisfaction [40]. Finally, consistent with the proposed mediation model (see Figure 1), we predicted that the relationship between EI and job satisfaction would be mediated by workers’ engagement after controlling for variance in age, gender and years of work.

## 2. Materials and Methods

### 2.1. Sample

We recruited a multi-occupational sample of 405 workers (56% women) from the general population. Participants’ ages ranged from 21 to 64 years (mean age = 40.72 years, standard deviation = 10.32 years, median = 42 years). The sample was a convenience sample, consisting of self-employed workers (41.7%), building and maintenance (11.4%), administration (11.1%), education (8.4%), human services (7.4%), commercial services (6.4%), health services (6.2%) and employees from a diverse range of other occupations (7.4%).

### 2.2. Measures

#### 2.2.1. Wong and Law Emotional Intelligence Scale

We used the Spanish version of the Wong and Law Emotional Intelligence Scale (WLEIS) [47,48] to measure perceived EI. This self-report measure is based on Salovey and Mayer’s [49] definition of EI and consists of 4 four-item subscales measuring: self-emotion appraisal (SEA), other-emotion appraisal (OEA), use of emotion (UOE) and regulation of emotion (ROE). Responses are given using a seven-point scale ranging from 1 (strongly disagree) to 7 (strongly agree). Sample items are: ‘I am sensitive to the feelings and emotions of others’ and ‘I am quite capable of controlling my own emotions.’ A global EI score is calculated; higher scores indicate greater EI. The Spanish version of the WLEIS has shown good validity and reliability in Spanish populations [48]. In this study Cronbach’s alpha coefficient for total EI was 0.90.

#### 2.2.2. Utrecht Work Engagement Scale

We used the Spanish version of the instrument [32,50]. This scale comprises 15 items measuring three aspects of work engagement: vigour (sample item: ‘I am bursting with energy in my work’), dedication (sample item: ‘I find my work full of meaning and purpose’) and absorption (sample item: ‘When I am working, I forget everything around me’). Responses are given using a seven-point scale ranging from 0 (never) to 6 (daily). In this study, the Cronbach’s alpha coefficients for the three subscales were: vigour = 0.83; dedication= 0.88 and absorption = 0.82.

#### 2.2.3. Overall Job Satisfaction Scale

Job satisfaction was measured using an overall job satisfaction scale taken from Judge et al. [51] which was based on the overall job satisfaction scale developed by Brayfield and Rothe [52]. The scale consists of five items to which responses are given using a seven-point scale ranging from 1 (strongly disagree) to 7 (strongly agree). It includes items such as ‘I am often bored with my job’ and ‘Most days I am enthusiastic about my work.’ Item scores are summed to give an index of overall measure of job satisfaction; higher scores indicate greater overall job satisfaction. The English scale has shown adequate reliability (0.88) and high convergent validity relative to a composite index based on facets of the Job Descriptive Index [53]. In this study, the scale showed adequate reliability (Cronbach’s alpha = 0.71).

### 2.3. Procedure

A student-recruited sampling [54] was used to collect data from participants, this allowed us to access a community sample from a university setting. In this sense, prior research has considered these sampling methods as a reliable technique with employed adults [55], thereby being used in recent studies with occupational samples [55,56]. Students enrolled in a psychology course at University were asked to recruit five adult workers at maximum through their personal network and administer the battery of questionnaires to them. The battery included a letter explaining the goal of the study and emphasising that responses would be anonymous and confidential and socio-demographic questions on age, sex, years of work and occupation, along with several questionnaires. Participants were asked to complete and return their questionnaires to university students.

Data collection was carried out in compliance with the principles defined in the Declaration of Helsinki. Participants were informed that participation in the study was voluntary and anonymous and verbal, informed consent was obtained. Students returned completed questionnaires to the teaching staff for statistical processing. The study protocol was approved as part of the project PSI2012-38813 by the Research Ethics Committee of the University of Malaga.

### 2.4. Data Analysis

Data were analysed using Statistical Package for the Social Sciences, SPSS version 23.0 (SPSS Inc., Chicago, IL, USA). After calculating means, standard deviations and reliabilities for the measured variables and computing the Pearson’s zero-order correlation coefficients among EI, engagement dimensions and job satisfaction, multiple mediation analyses were conducted for testing for potential mediators in the EI-job satisfaction link. We applied the bootstrapping method to a prediction model involving multiple mediators to calculate overall indirect effects and specific indirect effects. This procedure allows several indirect (i.e., mediated) effects in a model to be examined simultaneously (through the pathway for each mediator variable) as well as providing a measure of the direct effect of the predictor variable on the criterion variable, whilst accounting for potential collinearity among the mediator variables. Bootstrapping with 5000 re-samples was used to obtain parameter estimates for both overall and specific indirect effects. The 95% bias-corrected CIs were used to determine whether effects were statistically significant: if the 95% bias-corrected CI does not contain zero the indirect effect is considered statistically significant and mediation has been demonstrated [57]. To determine the relative magnitude of the three specific indirect effects we calculated contrasts for significant specific indirect effects using bias-corrected and accelerated bootstrap intervals. Similarly, if the 95% CI does not contain zero, this is taken as evidence of relevant importance of the individual mediators’ unique role in the association between EI and job satisfaction. To avoid the possibility that associations between EI and job satisfaction would be confounded by socio-demographic factors we also controlled for effects of age, sex and years of work.

## 3. Results

### 3.1. Descriptive Analyses

First, given that factors such as age are relevant in relation to job satisfaction [44], we examined participants’ ages in relation to their reported occupations (see Table 1). Second, we tested whether age groups could reflect significant differences in either engagement dimensions or job satisfaction.) We split the sample between young adults (18 to 35-year-old workers, *N* = 132) and middle-aged professionals (older than 35-year-old, *N =* 273). Results did not show significance for differences between groups regarding vigour (F = 1.261; *p* = 0.26), dedication (F = 2.99; *p* = 0.09), absorption (F = 2.71; *p* = 0.10), nor job satisfaction (F = 0.73; *p* = 0.39).

Lastly, we conducted descriptive analyses. Means, standard deviations, Pearson correlations and reliabilities for the study variables are shown in Table 2.

As Table 2 shows, EI was positively correlated to all three engagement variables (vigour: *r* = 0.46; dedication: *r* = 0.38; absorption: *r* = 0.33) as well as with job satisfaction (*r* = 0.35). As expected all three engagement variables were moderately positively associated with job satisfaction (vigour: *r* = 0.57; dedication: *r* = 0.68; absorption: *r* = 0.43).

### 3.2. Multiple Mediation Analyses

We examined a simultaneous mediation of the EI- job satisfaction by the three work engagement variables using, a multiple mediator model, using bias-corrected and accelerated bootstrapping to establish confidence intervals (CIs) [57].

**Figure 2:**
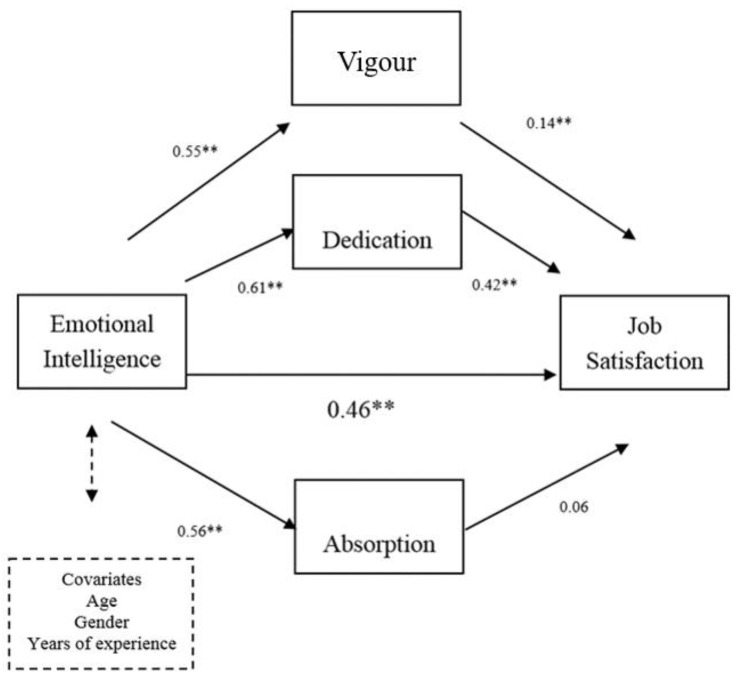
Multiple mediation model of the work engagement dimensions explaining the relationship between emotional intelligence and job satisfaction. ** *p* < 0.01.

Table 3 summarises the results of the multiple mediator analysis, giving the overall, direct and indirect effects and their 95% CIs. None of the covariate (age; gender; years of work) effects was significant (all *p*s > 0.10). As seen in Figure 2, bootstrap estimation showed that the overall effect of EI on job satisfaction was significant (*c* = 0.46; *p* < 0.01). When variance associated with the hypothesised mediators was controlled the association between EI and job satisfaction was no longer significant (*c*’ = 0.09; *p* > 0.10), suggesting that it was mediated by these variables. As shown in Table 3, vigour and dedication showed a significant indirect effect (indirect effect for vigour = 0.082; 95% CI = 0.011, 0.172; indirect effect for dedication = 0.259; 95% CI = 0.179, 0.349) but absorption did not (indirect effect = 0.034; 95% CI = −0.001, 0.079). We also examined the contrast between the significant indirect effects and found that the indirect effect of dedication was significantly greater than the indirect effect of vigour (C_1_ = −0.17; 95% CI = −0.314, −0.046), which suggests that dedication is the more important mediator of the EI-job satisfaction association. In summary, after controlling for effects of sex, age and years of work, dedication and vigour (to a lesser extent) completely mediated the relationship between EI and job satisfaction. Together the three mediating variables and covariates accounted for 47.09% of the variance in job satisfaction (R^2^ adj = 0.47; *F* (7, 395) = 52.94; *p* < 0.001).

Post hoc analyses were conducted separately for each EI dimension, showing a similar pattern than the above-mentioned findings. That is, the relationship between each EI dimension and job satisfaction was fully mediated by vigour and dedication, whereas absorption did not mediate this relationship (see Table 3).

## 4. Discussion

This study extends previous empirical research showing that EI [21] and work engagement [40] influenced job satisfaction. Our results also extend the findings of a recent meta-analysis [16], by showing that a positive motivational construct, such as work engagement, may underlie the association between EI and job satisfaction.

Consistent with prior work [21,24], we found that EI was positively associated with job satisfaction. Drawing on theories of dispositional sources of job satisfaction and Job Demands-Resources (JD-R) model, our results suggest that EI might be another key personal resource related to job satisfaction, along with well-known classic dimensions (i.e., affect, big five, core self-evaluations…) [4,58]. Therefore, several studies have emphasised that the ability to perceive, understand and regulate the emotions of oneself has an important relationship with job satisfaction [16,21]. Our findings lend additional support to these previous findings and provide insight into the joint contribution of emotional abilities and underlying motivational work-related processes to job satisfaction.

We also found that workers with higher EI also report higher dedication, vigour and absorption. These results confirm earlier research suggesting that EI is positively associated with work engagement in professionals [36,37,38]. Pena, Rey and Extremera [37] noted that workers with high EI seem to show higher vigour and energy at work, report higher enthusiasm, inspiration, pride and challenge in relation to work and show greater concentration and energy during job activity. Expanding this previous work, it is possible that workers with high EI experience greater work-related vigour, dedication and absorption and therefore, increased positive attitudes in the workplace.

On the other hand, our multiple mediation analyses indicated that EI has an indirect effect on job satisfaction, via vigour and dedication. In line with earlier work showing that work engagement is linked to job satisfaction [40,48], we found that the three engagement variables were associated with job satisfaction. Path analyses indicated that together vigour and dedication fully mediated the association between EI and job satisfaction but the indirect effect of dedication was significantly greater than that of vigour both for EI and its subdimensions. Our results showed that all dimensions comprising EI construct showed the same pattern of relations with engagement dimensions and job satisfaction, thereby suggesting they are equally important in this link. These findings are consistent with earlier research showing that work engagement mediated relationships between personal resources and several organisational outcomes [27,28,29,30]. Our results also extend previous research as they suggest that dedication is a more important mediator of the EI-job satisfaction association than vigour. However, our results did not provide evidence that absorption in work mediates the EI-job satisfaction link. One possible explanation for this is that absorption is a consequence of work engagement, rather than one of its components [59]. Vigour and dedication can be considered the core dimensions of engagement whereas absorption might be considered an outcome of vigour and dedication. Some authors have concluded that absorption is not a construct unique to work engagement; rather it overlaps with other unhealthy constructs such as workaholic [60]. Further research on this issue in work is recommended.

In general, employees with high emotional abilities typically experience more positive mood as well as having greater energy and willingness to invest to work [36]. Similarly, experiencing greater enthusiasm, inspiration and challenge whilst at work plays an important role in the development and maintenance of positive job attitude [40]. Hence, employees with high EI might have the natural dispositions to display positive and fulfilling work-related mood states compared to low EI counterparts. Finally, employees who are highly engaged in their work may tend to experience more positive job outcomes as commitment to the organisation and experience greater job satisfaction [61,62]. Confirmation of our findings in a longitudinal study would provide robust evidence that high EI employees tend to be more engaged in their work and hence display a more positive job attitude. In the same vein, examination of differences in job satisfaction by age, experience or occupations would be a fruitful avenue aiming at designing more effective interventions.

Our results make a contribute to the theoretical literature on EI and mediating process in organisational settings but they could also be used to inform the design of integrative preventions aimed at increasing employees’ energy and pride in their work, with a view to increasing positive job attitudes. Organisations should attempt to provide a supportive workplace environment and reduce workplace risks associated with negative job attitudes [2,44], occupational stress [63] and lower work engagement [64]; but training professionals in emotional skills might also increase employees’ energy, effort, enthusiasm and sense of significance in the workplace. Using personnel selection methods based on EI [65] or engagement [66] to ensure a good match between individual employees’ affective and motivational resources and job factors should increase their job satisfaction. Further research should seek to expand our findings by investigating whether the two theoretical constructs we have explored, EI and work engagement, also play a key role in the reduction of negative workplace outcomes such as sickness absence, turnover intention and deviant workplace deviant behaviour.

The contributions of this study should be assessed in the light of its limitations. As is common in the field, we used a self-report measure of EI but future studies should use EI performance tests such as Mayer-Salovey-Caruso Emotional Intelligence Test [67] to generalise our findings. Thus, as previous research has confirmed that self-report and ability EI differ in their conceptualization of EI and they also might differ in their predictive validity regarding personal and workplace outcomes [16,68], further exploration using both EI measurement approaches would allow researchers to examine potential peculiarities and implications of using each approach as well as the specific contribution of both EI assessment method in explaining work attitudes. Moreover, although our data provide evidence for our proposed mediation model, the cross-sectional nature of the data mean it is impossible to determine the direction of relationships between variables. In other words, it is possible that employees’ work engagement might influence their overall appraisal of their own emotional abilities. Replicating our findings in a longitudinal study would provide further insights to the causal relationships between EI, work engagement and job satisfaction. Besides, our study included workers recruited by student-recruited sampling, which is a non-random sampling technique. Although this technique is a valuable and reliable tool increasingly used in organisational research [56,69], the use of student-recruitment sampling methods in organisational research may be more biased toward the more cooperative workers participants who are willing to participate in the study, thereby limiting the generalization of our results. It is also important to underline that our participants were healthy adult workers and that our findings may not generalise to distressed adults or workers on sick leave. Further work with samples of employees on medically certified long-term sick leave would be useful. Finally, we used an indicator of overall job satisfaction and so we have no information about the relative strength of associations between EI and specific aspects of job satisfaction. Future research should examine the links between specific facets of job satisfaction and EI in order to provide insight into the different contributions of EI and engagement to specific intrinsic and extrinsic aspects of job characteristics.

Despite these limitations, our research provides further empirical evidence that EI should be considered a personal resource that is relevant to job satisfaction. Our empirically supported mediational model extends the EI literature by elucidating the pathways through which EI is linked with job satisfaction. Taken together, these results suggest that employees with high EI report higher vigour and dedication at work, which, in turn, increases their job satisfaction.

## 5. Conclusions

Since there are several routes to increased employee job satisfaction not just changes in working conditions but also changes in perception of one job- and job perception is influenced by the disposition of the individual worker, it is necessary to examine how these different routes might be promoted. In line with prior research, our findings suggest that EI interventions aimed at increasing work engagement and positive mood might be particularly useful in increasing job satisfaction.

## Figures and Tables

**Table 1:** Subject characteristics in relation to their occupation.

Career Groups	*N*	Age
Self-employed workers	169	41.17 (10.28)
Building and maintenance	46	37.63 (9.75)
Human services	30	31.93 (8.20)
Commercial services	26	43.96 (10.07)
Education	34	44.06 (8.84)
Public administration	45	43.98 (8.78)
Health services	25	47.08 (10.12)
Others	30	35.07 (8.75)

Note: *N* = 405.

**Table 2:** Descriptive statistics and correlations between study variables.

Variables	M	SD	α	1	2	3	4	5
1. Emotional intelligence	5.20	0.79	0.90	—				
2. Vigour	4.92	0.96	0.83	0.46 **	—			
3. Dedication	4.64	1.29	0.88	0.38 **	0.72 **	—		
4. Absorption	4.23	1.32	0.82	0.33 **	0.50 **	0.53 **	—	
5. Job satisfaction	4.96	1.07	0.71	0.35 **	0.57 **	0.68 **	0.43 **	—

Note: *N* = 405. ** *p* < 0.01. M = Mean; SD = Standard Deviation. α = Cronbach’s alpha.

**Table 3:** Test of the mediating effect of work engagement dimensions.

Model Pathways	Point Estimate	SE	Normal Theory Tests			95% Bias-Corrected CI	
			Effect	*Z*	*p*	Lower	Upper
Total effect	0.376	0.05				0.284	0.481
EI → Vigour → Job satisfaction	0.082	0.04	0.08	2.28	*p* < 0.05	0.011	0.172
EI → Dedication → Job satisfaction	0.259	0.04	0.25	6.07	*p* < 0.01	0.179	0.349
EI → Absorption → Job satisfaction	0.034	0.01	0.03	1.63	*p* = 0.10	−0.001	0.079
Model F (7, 395) = 52.94; *p* < 0.001; R^2^ = 0.48; R^2^ adj = 0.47							
Contrasts for significant indirect effects							
C_1_ = Vigour vs. Dedication	−0.17	0.06				−0.314	−0.046
Total effect	0.264	0.04				0.176	0.362
SEA → Vigour → Job satisfaction	0.060	0.03	0.06	2.32	*p* = 0.02	0.006	0.128
SEA → Dedication → Job satisfaction	0.179	0.03	0.17	5.07	*p* < 0.01	0.111	0.264
SEA → Absorption → Job satisfaction	0.024	0.01	0.02	1.65	*p* = 0.09	0.001	0.059
Model F (4, 398) = 10.52; *p* < 0.001; R^2^ = 0.09; R^2^ adj = 0.15							
Total effect	0.237	0.04				0.1585	0.322
OEA → Vigour → Job satisfaction	0.057	0.02	0.05	2.51	*p* = 0.01	0.014	0.114
OEA → Dedication → Job satisfaction	0.159	0.03	0.15	4.72	*p* < 0.01	0.101	0.237
OEA → Absorption → Job satisfaction	0.021	0.01	0.02	1.68	*p* = 0.09	0.003	0.049
Model F (4, 398) = 6.89; *p* < 0.001; R^2^ = 0.06; R^2^ adj = 0.12							
Total effect	0.271	0.04				0.194	0.358
UOE → Vigour → Job satisfaction	0.061	0.03	0.06	2.15	*p* = 0.03	−0.001	0.127
UOE → Dedication → Job satisfaction	0.184	0.03	0.18	5.49	*p* < 0.01	0.123	0.258
UOE → Absorption → Job satisfaction	0.026	0.01	0.02	1.59	*p* = 0.11	−0.001	0.060
Model F (4, 398) = 13.37; *p* < 0.001; R^2^ = 0.20; R^2^ adj = 0.11							
Total effect	0.228	0.03				0.160	0.303
ROE → Vigour → Job satisfaction	0.049	0.02	0.04	2.52	*p* = 0.01	0.010	0.096
ROE → Dedication → Job satisfaction	0.156	0.03	0.15	5.50	*p* < 0.01	0.102	0.225
ROE → Absorption → Job satisfaction	0.022	0.01	0.02	1.68	*p* = 0.09	0.001	0.051
Model F (4, 398) = 10.31; *p* < 0.001; R^2^ = 0.14; R^2^ adj = 0.09							

Note: *N* = 405. SE= Standard Error. CI = Confidence Interval. EI = Emotional Intelligence. SEA = self-emotion appraisal. OEA = other-emotion appraisal. UOE = use of emotion. ROE = regulation of emotion.
